# Enasidenib‐induced hepatitis in an individual with Type II D2‐hydroxyglutaric aciduria

**DOI:** 10.1002/jmd2.12421

**Published:** 2024-04-16

**Authors:** Jessica I. Gold, Arianna K. Stefanatos, Jamie L. Fraser, Adeline Vanderver, Sanmati Cuddapah

**Affiliations:** ^1^ Division of Medical Genetics, Department of Pediatrics Northwell Health New York New York USA; ^2^ Department of Child and Adolescent Psychiatry and Behavioral Sciences Children's Hospital of Philadelphia Philadelphia Pennsylvania USA; ^3^ Prenatal Pediatrics Institute Children's National Hospital Washington DC USA; ^4^ The George Washington University School of Medicine and Health Sciences Washington DC USA; ^5^ Division of Fetal and Translational Medicine Children's National Hospital Washington DC USA; ^6^ Division of Neurology, Department of Pediatrics Children's Hospital of Philadelphia Philadelphia Pennsylvania USA; ^7^ Department of Neurology Perelman School of Medicine, University of Pennsylvania Philadelphia Pennsylvania USA; ^8^ Section of Biochemical Genetics, Division of Genetics, Department of Pediatrics Children's Hospital of Philadelphia Philadelphia Pennsylvania USA

**Keywords:** IDH2 inhibitor, novel treatment, organic acidurias, Type II D2‐hydroxyglutaric aciduria

## Abstract

Type II D‐2‐Hydroxyglutaric aciduria (T2D2HGA) is caused by a gain‐of‐function pathogenic variant in Isocitrate Dehydrogenase 2 (IDH2). Patients with T2D2HGA commonly present with developmental delay, seizures, cardiomyopathy, and arrhythmias. The recently approved IDH2‐inhibitor Enasidenib targets the p.Arg140Gln pathogenic *IDH2* variant and decreases production of D2HGA. We present a 7‐year‐old female with T2D2HGA due to the p.Arg140Gln variant. She was diagnosed at 3‐years‐old after presenting with global developmental delay, leukoencephalopathy, communicating hydrocephalus, seizures, and dilated cardiomyopathy. At age 3 years 11 months, 50 mg Enasidenib daily was initiated. Primary outcomes included seizure frequency, hospital admissions, development, and cardiac structure. Laboratories were monitored biweekly for common Enasidenib side effects. Our patient tolerated Enasidenib well. Urine 2‐HGA decreased significantly from 244 mg/g creatinine to undetectable within 2 weeks of treatment. Inpatient admissions decreased from 8 during the 2 years preceding treatment to 1 during treatment. She has been seizure‐free since Enasidenib initiation. Echocardiography showed improvement in dilated cardiomyopathy with normal left ventricular systolic function. Developmental assessment demonstrated improvements in gross motor, fine motor, language, and socialization domains. Treatment was complicated by mild elevations in alanine transaminase (118 IU/L, range 0–28) and creatine kinase (334 U/L, range 45–198) that resolved by decreasing Enasidenib dosing frequency to three times weekly. Enasidenib is a viable treatment for Type II D2HGA with benefits including developmental gains, fewer acute medical interventions, and cardiomyopathy improvement. While drug‐induced hepatitis is a novel adverse effect of Enasidenib, it can be ameliorated by decreasing dose frequency.

**Synopsis**

Enasidenib, a targeted IDH‐2 inhibitor, can be used for treatment of Type II D2‐hydroxyglutaric aciduria. Clinicians should monitor liver function tests for potential adverse effects.

## 1 INTRODUCTION

**FIGURE 1:**
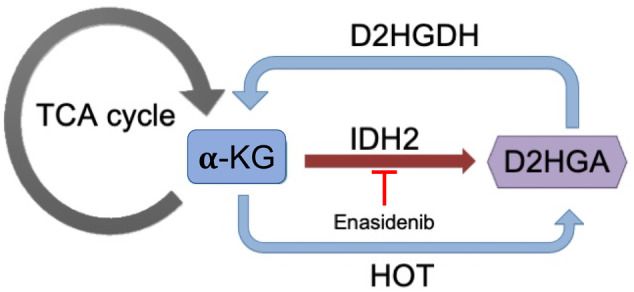
Schematic of the production of D2‐hydroxyglutaric acid (D2HGA) in Type II D2HGA and the mechanism of action of Enasidenib. ɑ‐ketoglutarate (α‐KG) is an intermediate in the citric acid (TCA) cycle and the substrate for the formation of D2HGA through Isocitrate Dehydrogenase 2 (IDH2) or hydroxyacid‐oxoacid transhydrogenase (HOT). Type II D2HGA is an autosomal dominant organic aciduria, caused by a heterozygous gain‐of‐function variant in IDH2 (p.Arg140Gln) that preferentially catalyzes the formation of D2HGA. Enasidenib is a targeted inhibitor against IDH2 p.Arg140Gln that decreases D2HGA production. By contrast, Type I D2HGA is an autosomal recessive disorder caused by loss‐of‐function variants in D2HGA dehydrogenase (D2HGADH), which converts D2HGA to α‐KG.

Type II D2‐hydroxyglutaric aciduria (D2HGA) is a rare, autosomal dominant, progressive neurometabolic disorder. It is caused by a gain‐of‐function pathogenic variant in *IDH2*, which leads to increased production by isocitrate dehydrogenase 2 (IDH2) of D‐2‐hydroxyglutarate (D2HG) from alpha‐ketoglutarate (a‐KG).^1^, ^2^ D2HGA is converted back to 2‐KG via D2HGA dehydrogenase (D2HGADH), a flavin‐dependent enzyme and the causative enzyme of the autosomal recessive‐inherited Type I D2HGA (Figure 1).^1^ There is no known physiological use of D2HG and it is likely toxic to neurons and other cells.^3^

Approximately 20 individuals with Type II D2HGA have been described in the literature. All have developmental delay that manifest by 2 years old.^4^ Epilepsy is common, occurring in over 80% of reported patients. Nearly half of the published patients also have cardiomyopathy, a feature unique to Type II D2HGA. The oldest described patient lived to 22 years old, and currently living patients range from infancy to 19 years old.^4^ Abnormal MRI findings have been reported including delayed myelination with open opercula.^5^

Current treatment for type II D2HGA is supportive. Riboflavin supplementation may optimize the function of D2HGADH, leading to increased conversion of D2HG to α‐KG.^6^ However, a targeted IDH2‐inhibitor, Enasidenib, has recently been approved for use in refractory acute myeloid leukemia (AML).^7^ Enasidenib specifically targets the p.Arg140Gln pathogenic *IDH2* variant and decreases production of D2HG.^8^ Enasidenib use has recently been reported in two individuals with Type II D2HGA.^9^ These individuals demonstrated gains in developmental milestones and stabilization of cardiomyopathy with only hyperbilirubinemia as a side effect.^9^ Here, we support the benefits of Enasidenib treatment in Type II D2HGA while also reporting drug‐induced hepatitis as a novel adverse effect.

## 2 CASE

Our patient is 7 year‐old female with Type II D2HGA due to the p.Arg140Gln pathogenic *IDH2* variant. She was born via cesarean section at 43 weeks gestational age to a non‐consanguineous couple. APGAR scores were 3 at 5 min and 9 at 10 min. Her newborn screen was normal. A murmur detected in the newborn nursery prompted cardiology evaluation during her first month. While the murmur was deemed benign, an electrocardiogram (EKG) showed a borderline prolonged QTc interval at 455 ms.

Her early infancy is notable for repeat visits to the pediatrician for fussiness and poor feeding that improved after starting ranitidine. At 4.5 months old, our patient presented to her local emergency department for episodes of generalized twitching occurring 3–5 times daily. She was noted to have hypotonia and cortical visual impairment with stellate irises. Electroencephalogram (EEG) was disorganized with scattered sharp activity. She was started on Levetiracetam.

At 6 months, she presented to the leukodystrophy clinic at the Children's Hospital of Philadelphia for evaluation of seizures and gross and fine motor delay. Our patient did not roll, sit unsupported, and continued to have head lag when pulled to sit. She rarely grasped for objects, reached, or transferred between hands. Her exam was notable for generalized hypotonia, failure to track, and fisted hands. Her growth parameters were 6.85 kg (20%) for weight, 69 cm (80%) for length, and 43.5 cm (65%) for head circumference. Laboratories recommended at that time (CSF cell count and neurotransmitters, plasma very long chain fatty acids) were normal. Whole genome sequencing, conducted on a research basis, was non‐diagnostic. Our patient underwent brain MRI at 11 months old that showed under myelination suggestive of an underlying leukodystrophy and diffuse cerebral volume loss. A ventriculoperitoneal shunt was placed at 11 months for hydrocephalus that presented as vomiting, sleepiness, and papilledema. Oxcarbazepine was added for recurrent seizures.

Re‐evaluation in the leukodystrophy clinic at 15 months showed slow improvements in gross motor and fine motor skills, though well below expectations for age. She was able to roll and sit with propping for 4–5 s. She began grasping objects and transferring them between hands. She remained non‐verbal but would giggle in response to her parents. She ate entirely by mouth. From ages 1 year to 3 years, her weight gain and linear growth velocity remained stable at the 5th% and 10th%, respectively. However, measurements of her head circumference demonstrated microcephaly (0.2th%). She had six admissions for febrile seizures, vomiting, and feeding intolerance. Repeat EKGs showed increasingly prolonged QTc intervals to 546 ms. Echocardiography at age 3 years showed low normal left ventricular systolic shortening and mild left ventricular dilatation. During an admission in December 2019 at age 3 years, urine organic acids were analyzed by tandem mass spectroscopy due to concern for an underlying inherited metabolic disorder. They returned with markedly increased excretion of 2‐hydroxyglutarate and 2‐hydroxyglutaryllactone. Genetic testing for 2‐hydroxyglutaric acidurias returned with a de novo pathogenic variant in *IDH2*, c.419G > A (p.Arg140Gln), confirming the diagnosis of Type II D2HGA. Medical history coincided with key reported features of Type II D2HGA, including severe global developmental delay, microcephaly, epilepsy, cyclic vomiting, and cardiomyopathy.^4^, ^5^ Her brain MRI showing delayed cerebral maturation and diffuse volume loss was consistent with previously reported imaging in D2HGA.^10^ Treatment of D2HGA is supportive. Our patient was started on daily riboflavin at 100 mg to augment the activity of D2HGDH and increase conversion of D2HG to 2‐KG.

At age 3 years and 11 months, we initiated daily Enasidenib treatment. Enasidenib is an IDH2 inhibitor that specifically targets the p.Arg140Gln variant and inhibits D2HG production. It has been approved for treatment of refractory acute myelogenous leukemia (AML) in adults and is currently in clinical trials for use in pediatric patients.^11^, ^12^ Enasidenib is an oral medication that is dosed daily based on body surface area. The most common adverse effects are unconjugated bilirubinemia, non‐infectious leukocytosis, and nausea. Differentiation syndrome has been reported in Enasidenib‐treatment individuals with AML.^13^ This syndrome, caused by rapid maturation of immature myeloid cells, presents with fever, pericardial and pleural effusions, hypotension, and renal failure. It has been observed between 10 days and 15 months after Enasidenib initation.^14^ As our patient did not have a large pool of immature myeloid cells, the risk of differentiation syndrome was deemed very low.

Enasidenib was dosed at 50 mg daily. Within 24 h of medication initiation, urine organic acid analysis showed 84% decrease in D2HG (Table 1). We monitored our patient with biweekly complete metabolic panels and complete blood counts for the first 6 months. These laboratories were then spaced to every 2–3 months. There was no elevation in her unconjugated bilirubin or white blood cell count through the initiation period. Urine organic acid analyses were sent bimonthly and continued to ensure suppression of D2HG production.

**TABLE 1:** Laboratory values pre‐ and post‐Enasidenib Initiation.

	Diagnosis	Enasidenib initiation	Enasidenib treatment: 24 h	Enasidenib treatment: 1 month	Enasidenib treatment: 6 months	Enasidenib treatment: 12 months	Enasidenib treatment: 18 months	Enasidenib treatment: 24 months	Enasidenib treatment: 30 months	Reference range
Chemistry										
Sodium	139	136	134	140	144	140	140	144	139	134–144 mmol/L
Potassium	3.3	3.9	4.2	4.3	3.6	3.8	4.6	4.2	3.3	3.5–5.2 mmol/L
Chloride	105	102	100	105	106	102	104	103	97	96–106 mmol/L
Carbon dioxide	20	24	25	23	23	24	20	20	24	17–26 mmol/L
Urea nitrogen	6	20	21	23	14	9	6	8	10	5–18 mg/dL
Creatinine	<0.2	<0.2	0.2	0.29	0.38	0.34	0.37	0.43	0.43	0.3–0.59 mg/dL
Glucose	81	88	76	54	93	83	94	79	109	65–99 mg/dL
Calcium	9.5	10.4	10.5	10.2	9.4	9.9	10.5	10.5	9.8	9.1–10.5 mg/dL
Total Bilirubin	0.3	0.3	0.4	0.4	0.6	0.5	0.3	<0.2	0.3	0.0–1.2 mg/dL
Total Protein	6.8	7.2	7.3	6.6	6.4	6.7	6.9	6.7	6.0	6.0–8.5 g/dL
Albumin	3.7	4.3	4.4	4.4	4.6	4.6	4.5	4.7	3.7	4.0–5.0 g/dL
Alkaline phosphatase	88	148	156	179	140	160	166	175	104	158–369 IU/L
Alanine aminotransferase	11	24	23	42	**118**	**62**	**31**	25	31	0–28 IU/L
Aspartate aminotransferase	26	49	31	39	**60**	40	31	29	33	0–60 IU/L
GGT	14				17	14	12			0–60 IU/L
Amylase	<30									
Lipase	77									
CK					**334**	**297**	**240**	**290**		45–198 U/L
Hematology										
White blood cell count		9.2	10.4	8.8	7.8	**15.3**	11.2	11.2	10.8	4.3–12.4 × 10^3/μL
Hemoglobin		11.9	11.9	12.2	12.4	12	12.5	12.8	11.7	10.9–12.4 g/dL
Hematocrit		35.5	35.8	37.1	37.1	34.5	36.9	39.2	35.2	32.4–43.4%
Platelet		367	362	384	364	348	400	389	274	150–450 × 10^3/μL
Metabolic										
2‐Hydroxyglutaric acid	**364**	**244**	**23**	0	0	0	0	0	17	(mg/g creatinine)

*Note*: Abnormal values are bolded.

Abbreviations: CK, creatine kinase; GGT, gamma‐glutamyl transpeptidase.

Interval evaluation occurred at 15, 30, and 36 months post‐initiation. Now 7 years old, our patient displayed improved gross motor skills including unsupported sit for 3–4 min, crawling, pulling to stand, and cruising. She is learning to use an assistive communication device and will bring food to her mouth for self‐feeding. Clinical neuropsychology assessment using the Bayley‐4 and Vineland‐3 was conducted prior to Enasidenib initiation and at 15 and 36 months post‐Enasidenib initiation. Gross motor skills improved from 4 month to 10 month age equivalents on both assessments, while fine motor skills improved from 6 month to 9 month age equivalents. Both assessments shows receptive language improvements from 1 month to 10 month age equivalents, and expressive language improvements from 4 month to 10 month age equivalents. Socialization reported by the Vineland‐3 improved from 1 month to 9 month age equivalents. Cognitive and play skills were stable at 6 month age equivalents. Repeat echocardiography showed resolution of left ventricular dilation to normal measurements, and normal cardiac function. EKG showed normalized QTc length to 430 ms. Repeat brain MRI was not obtained due to parental preference to avoid anesthesia. She remained seizure‐free and was weaned off her anti‐epileptic medications. She also required a significant decrease in acute care utilization. Since starting Enasidenib, our patient had one hospital admission in the last 36 months compared to eight in the prior 36 months. She had one emergency department visit that did not lead to admission compared to five visits in the 3 years prior.

At 18 months post‐initiation, a persistent mild elevation in alanine transferase (ALT) was noted on liver function tests (Table 1). Right upper quadrant ultrasound showed a mildly echogenic liver which could be related to steatosis or medications. Laboratories for liver dysfunction were performed, including coagulation studies, GGT, TSH, viral hepatitis panel, iron profile, ceruloplasmin, celiac panel, and ANA. Of this testing, only creatinine kinase (CK) returned elevated at 334 U/L (reference range 45–198 U/L). Due to concern for toxicity, the frequency of Enasidenib dosing was decreased from daily to three times weekly. Liver function tests completed 12 weeks after the dose adjustment showed normalization of ALT to 31 IU/L and CK to 240 IU/L. Repeat analysis of urine organic acids on the three times weekly Enasidenib dosing schedule continued to show full suppression of D2HG. Attempts to further decrease dose frequency to twice weekly were unsuccessful with reappearance of D2HG in urine organic acids analyzed as a trough sample.

## 3 DISCUSSION

Our experience with Enasidenib further supports its use as treatment for Type II D2HGA. Enasidenib is an easily administered oral medication. It successfully suppressed the production of D2HGA. During her 2 years of Enasidenib treatment, our patient has demonstrated developmental gains, especially in gross motor, fine motor, and language skills as assessed by the Bayley‐4 and Vineland‐3. Her Vineland‐3 assessment also showed improvement in socialization from 2 month to 10 month age equivalents, a significant milestones for parents who note her greater interactivity. Her seizures and cyclic vomiting episodes have ceased. In the last 3 years on Enasidenib, she has required acute medical intervention twice (one emergency room visit and one hospital admission), compared to the proceeding 3 years where she had three emergency room visits and eight inpatient admissions. Additionally, echocardiography demonstrated resolution of left ventricular dysfunction and normal cardiac function. Brain imaging has not been repeated since Enasidenib initiation due to parental preference and lack of clinical indication.

Drug‐induced hepatitis and liver imaging with mild, diffuse echogenicity has not previously been reported as a side effect of Enasidenib use. Enasidenib is manufactured as either a 50 or 100 mg tablet and dosed according to body surface area (BSA).^11^ To our knowledge, our patient is the youngest individual to be treated with Enasidenib. Her BSA at treatment initiation was 0.54 m^2^, well below the recommended BSA for the 50 mg tablet (1.3 m^2^). This mismatch may have increased the risk for drug toxicities. Laboratory improvements were noted with intermittent dosing (three times weekly), an adjustment that did not compromise D2HGA suppression or developmental gains. Pediatric formulations of Enasidenib may prevent recurrence of drug‐induced hepatitis in younger patients. This is important to note as Enasidenib should be initiated at the earliest convenience, and safe delivery to infants is necessary. Our monitoring did not detect any previously‐reported adverse effects of Enasidenib, including unconjugated hyperbilirubinemia, non‐infectious leukocytosis, or differentiation syndrome. This is likely related to the significant difference in treating an individual with a germline *IDH2* variant versus individuals with AML. Lacking a large clonal leukocyte population, our patient was not at high risk for myelogenous cell maturation and death.

The long‐term effects of Enasidenib treatment are unknown. Enasidenib was developed for use in acute refractory AML. The average Enasidenib treatment period in AML is 6 months.^15^, ^16^ Individuals with Type II D2HGA will likely require indefinite Enasidenib treatment. Intermittent dosing may alleviate adverse effects from life‐long Enasidenib treatment. Periodic monitoring of liver function and CK will be necessary. We are currently obtaining plasma laboratories (complete metabolic panel, complete blood count, coagulation studies, and CK) every 3 months. Urine organic acids are obtained at the same time and timed as trough measurements. Liver imaging has not been repeated due to improvements in our patient's ALT but will be considered for any concerning symptoms or signs of worsening liver function markers.

Enasidenib use for Type II D2HGA is a novel example of precision medicine in inherited metabolic disorders. Precision medicine‐based treatment is quickly becoming a pillar of treatment in oncology, rheumatology, and cardiology.^17^, ^18^, ^19^ These newly developed therapeutics are now being utilized as treatments for individuals with germline variants in disorders such as PIK3CA or familial hyperlipidemia.^20^, ^21^ To our knowledge, Enasidenib is the first application of a variant‐targeted pharmaceutical in an organic acidemia. Enasidenib may also be a treatment option for Type I D2HGA and combined D2, L2HGA, despite different molecular mechanisms. Ability to suppress D2HGA production through IDH2 may alleviate symptoms and improve neurodevelopmental outcomes.

## 4 CONCLUSION

Enasidenib, a targeted IDH2 inhibitor, is viable treatment for Type II D2HGA. Suppression of D2HGA production occurred within days of Enasidenib initiation. In the 3‐year period on Enasidenib, our patient reached new developmental milestones in gross motor, fine motor, receptive language, expressive language, and socialization domains. She required less emergent medical care and had few inpatient admissions. The primary adverse effect was medication‐induced hepatitis, which was ameliorated by intermittent Enasidenib dosing without sacrificing any benefits. Enasidenib should be considered a first‐line therapy for Type II D2HGA.

## AUTHOR CONTRIBUTIONS

All authors have participated in clinical assessments and care of the patient. JG and SC developed and initiated the Enasidenib treatment plan. JG drafted the initial manuscript. All authors participated in revision and approval of the final draft.

## FUNDING INFORMATION

This work was supported by NIH T32 GM008683.

## CONFLICT OF INTEREST STATEMENT

JG, AS, JF, and SC declare that they have no conflict of interest. AV receives research funding with no personal support from Biogen, Boehringer Ingelheim, Eli Lilly, Synaptix Bio, Illumina, Orchard, Sanofi, Ionis, Takeda, Affinia Therapeutics, Passage Bio.

## ETHICS STATEMENT

All procedures followed were in accordance with the ethical standards of the responsible committee on human experimentation (institutional and national) and with the Helsinki Declaration of 1975, as revised in 2000 (5). If doubt exists whether the research was conducted in accordance with the Helsinki Declaration, the authors must explain the rationale for their approach, and demonstrate that the institutional review body explicitly approved the doubtful aspects of the study.

## INFORMED CONSENT

Informed consent was obtained from all patients for being included in the study. Proof that informed consent was obtained must be available upon request.

## Data Availability

Deidentified data is available upon request to the corresponding author.

## References

[1] Saudubray J‐M , Baumgartner MR , Walter J . Inborn Metabolic Diseases. Springer; 2016. doi:10.1007/978-3-662-49771-5

[2] Kranendijk M , Struys EA , van Schaftingen E , et al. IDH2 mutations in patients with D‐2‐hydroxyglutaric aciduria. Science. 2010;330(6002):336. doi:10.1126/science.1192632 20847235

[3] Struys EA . D‐2‐Hydroxyglutaric aciduria: unravelling the biochemical pathway and the genetic defect. J Inherit Metab Dis. 2006;29(1):21‐29. doi:10.1007/s10545-006-0317-9 16601864

[4] Kranendijk M , Struys EA , Salomons GS , Van der Knaap MS , Jakobs C . Progress in understanding 2‐hydroxyglutaric acidurias. J Inherit Metab Dis. 2012;35(4):571‐587. doi:10.1007/s10545-012-9462-5 22391998 PMC3388262

[5] Srinivasaraghavan R , Sharma S , Kratz L , et al. Child with D‐2‐hydroxyglutaric aciduria type II: a rare neurometabolic disorder. Ann Indian Acad Neurol. 2021;24(6):933‐934. doi:10.4103/aian.AIAN_231_20 35359529 PMC8965948

[6] Yilmaz K . Riboflavin treatment in a case with l‐2‐hydroxyglutaric aciduria. Eur J Paediatr Neurol EJPN Off J Eur Paediatr Neurol Soc. 2009;13(1):57‐60. doi:10.1016/j.ejpn.2008.01.003 18343698

[7] Stein EM , DiNardo CD , Pollyea DA , et al. Enasidenib in mutant IDH2 relapsed or refractory acute myeloid leukemia. Blood. 2017;130(6):722‐731. doi:10.1182/blood-2017-04-779405 28588020 PMC5572791

[8] Amatangelo MD , Quek L , Shih A , et al. Enasidenib induces acute myeloid leukemia cell differentiation to promote clinical response. Blood. 2017;130(6):732‐741. doi:10.1182/blood-2017-04-779447 28588019 PMC5553578

[9] Geoerger B , Schiff M , Penard‐Lacronique V , et al. Enasidenib treatment in two individuals with D‐2‐hydroxyglutaric aciduria carrying a germline IDH2 mutation. Nat Med. 2023;29:1358‐1363. doi:10.1038/s41591-023-02382-9 37248298

[10] van der Knaap MS , Jakobs C , Hoffmann GF , et al. D‐2‐hydroxyglutaric aciduria: further clinical delineation. J Inherit Metab Dis. 1999;22(4):404‐413. doi:10.1023/a:1005548005393 10407777

[11] Kim ES . Enasidenib: first global approval. Drugs. 2017;77(15):1705‐1711. doi:10.1007/s40265-017-0813-2 28879540

[12] Enasidenib for the treatment of relapsed or refractory acute myeloid leukemia patients with an IDH2 mutation. Accessed September 7, 2022 https://clinicaltrials.gov/ct2/show/NCT04203316

[13] Fathi AT , DiNardo CD , Kline I , et al. Differentiation syndrome associated with Enasidenib, a selective inhibitor of mutant Isocitrate dehydrogenase 2: analysis of a phase 1/2 study. JAMA Oncol. 2018;4(8):1106‐1110. doi:10.1001/jamaoncol.2017.4695 29346478 PMC5885269

[14] Patel SA . Enasidenib‐induced differentiation syndrome in IDH2‐mutant acute myeloid leukemia. JAMA Oncol. 2018;4(8):1110‐1111. doi:10.1001/jamaoncol.2017.4724 29346477

[15] Pollyea DA , Tallman MS , de Botton S , et al. Enasidenib, an inhibitor of mutant IDH2 proteins, induces durable remissions in older patients with newly diagnosed acute myeloid leukemia. Leukemia. 2019;33(11):2575‐2584. doi:10.1038/s41375-019-0472-2 30967620 PMC9724489

[16] Reed DR , Elsarrag RZ , Morris AL , Keng MK . Enasidenib in acute myeloid leukemia: clinical development and perspectives on treatment. Cancer Manag Res. 2019;11:8073‐8080. doi:10.2147/CMAR.S162784 31564968 PMC6724422

[17] Waarts MR , Stonestrom AJ , Park YC , Levine RL . Targeting mutations in cancer. J Clin Invest. 2022;132(8):e154943. doi:10.1172/JCI154943 PMC9012285 35426374

[18] Burmester GR , Pope JE . Novel treatment strategies in rheumatoid arthritis. Lancet (London, England). 2017;389(10086):2338‐2348. doi:10.1016/S0140-6736(17)31491-5 28612748

[19] Currie G , Delles C . Precision medicine and personalized medicine in cardiovascular disease. Adv Exp Med Biol. 2018;1065:589‐605. doi:10.1007/978-3-319-77932-4_36 30051409

[20] Pagliazzi A , Oranges T , Traficante G , et al. PIK3CA‐related overgrowth spectrum from diagnosis to targeted therapy: a case of CLOVES syndrome treated with Alpelisib. Front Pediatr. 2021;9:732836. doi:10.3389/fped.2021.732836 34568242 PMC8459713

[21] Chen R , Lin S , Chen X . The promising novel therapies for familial hypercholesterolemia. J Clin Lab Anal. 2022;36(7):e24552. doi:10.1002/jcla.24552 35712827 PMC9279988

